# Global Electricity Trade Network: Structures and Implications

**DOI:** 10.1371/journal.pone.0160869

**Published:** 2016-08-09

**Authors:** Ling Ji, Xiaoping Jia, Anthony S. F. Chiu, Ming Xu

**Affiliations:** 1 School of Economics and Management, Beijing University of Technology, Beijing, China; 2 School of Environment and Safety Engineering, Qingdao University of Science & Technology, Qingdao, China; 3 Department of Industrial Engineering, De La Salle University, Manila, Philippines; 4 School of Natural Resources and Environment, University of Michigan, Ann Arbor, Michigan, United States of America; 5 Department of Civil and Environmental Engineering, University of Michigan, Ann Arbor, Michigan, United States of America; 6 Sustainable Development and New-Type Urbanization Think Tank, Tongji University, Shanghai, 200092, China; Beihang University, CHINA

## Abstract

Nations increasingly trade electricity, and understanding the structure of the global power grid can help identify nations that are critical for its reliability. This study examines the global grid as a network with nations as nodes and international electricity trade as links. We analyze the structure of the global electricity trade network and find that the network consists of four sub-networks, and provide a detailed analysis of the largest network, Eurasia. Russia, China, Ukraine, and Azerbaijan have high betweenness measures in the Eurasian sub-network, indicating the degrees of centrality of the positions they hold. The analysis reveals that the Eurasian sub-network consists of seven communities based on the network structure. We find that the communities do not fully align with geographical proximity, and that the present international electricity trade in the Eurasian sub-network causes an approximately 11 million additional tons of CO_2_ emissions.

## Introduction

In 2011, electricity generation contributed 19% of global primary energy use [1] and 42% of global CO_2_ emissions. The electricity generation industry was traditionally a tightly regulated sector, if not a national monopoly. Increasingly liberalized electricity markets worldwide [2] enable open access and free transit for international electricity exchanges, enabling trade in electricity between nations. Cross-border electricity trade can increase power plants’ effective capacity factor, enable a more diversified portfolio of generated resources, and improve the stability of individual grids [3]. For instance, the internal energy market (IEM) policy proposed the concept of the “European Supergrid” consisting of an integrated power system to better balance electricity supply and demand [4, 5]. However, cross-border electricity trade can increase uncertainty and risk to transmission capacities and local electricity systems, and thus such electricity trade requires special attention to ensure a reliable interconnected electricity network [6]. It is therefore crucial to identify critical national grids to enhance the stability of the global power grid, which requires an understanding of the structure of the global power grid.

Network analysis is widely used to uncover structural features of complex systems [7], with wide use in many fields such as scientific collaboration [8], biology [9, 10], food web [11], transportation [12–14], economics [15–19], social networks [9, 20], and environmental networks [21–24]. It can identify the role of nodes, discover communities, and predict a network’s future evolution. For example, Saracco *et al*. (2015) found that the behavior of the World Trade Web differs significantly from the monopartite analogue, showing highly non-trivial patterns of self-organization [25]. Vidmer *et al*. (2015) predicted the future evolution of international trade networks though link prediction algorithms [26]. Network analysis was recently introduced to analyze the structure of the global energy product trade network. For instance, Ji *et al*. (2014) analyzed the overall features, regional characteristics, and stability of the oil trade network, finding that it displays a scale-free behavior [27]. Zhang *et al*. (2014) found increasing intensity in the competition in the global oil trade [28]. Moreover, other studies focused on analyzing the structure of regional power grids to evaluate vulnerabilities related to cascading failures and intentional attacks [29–31]. However, there is no study, to the best of our knowledge, examining the structure of interconnected grids at the global scale, which facilitates identification of national grids critical for stability.

This is the first study to analyze the structural features of the global power grid from the perspective of a global electricity trade network, in which nations are nodes linked via international electricity exchanges. This study offers two main contributions. First, it analyzes the basic features and evolution of the global electricity trade network in terms of basic properties, important nodes (i.e., nations), and community structure with special focus on the largest Eurasian sub-network. Second, it evaluates the CO_2_ implications of this global electricity trade network in terms of CO_2_ mitigation.

## Methods and Data

### Network analysis

The global electricity trade network is weighted (i.e., each nation has a value of electricity imports and/or exports) and directed (i.e., electricity trade from nation A to nation B differs from that of nation B to nation A). Assume there are *n* nodes (nations) connected by *l* links (i.e., international electricity exchanges). The adjacency matrix $W_{D}^{t}$ represents the global electricity trade network, where $w_{D}^{t}\left(i,j\right)$ represents the volume of electricity trade from nation *i* to nation *j* in year *t*.

We use the following metrics to show the global electricity trade network structure: node degree, betweenness centrality, cluster coefficient, and community structure.

**Node degree** is one of the most common metrics in evaluating the importance of nodes in a network by counting its nearest neighbors. The **Node in-degree** of a particular nation counts the import relationship with other nations measured by total number of links from other nations to the focal nation. The **Node out-degree** counts the export relationship with other nations measured by total number of links from the focal nation to other nations. **Node strength** is an extended definition of node degree that adds the weights of links with its nearest neighbors, and measures the total weight of its connected links. **Average nearest-neighbor degree (*K***_**nn**_**)**, defined as the average degree of the nearest neighbor for vertices with degree *k*, is an important index analyzing network assortativity. We also define here the **average nearest-neighbor strength (*S***_**nn**_**)** as the average strength of the nearest neighbor for vertices with degree *k*.

Node degree only reflects the importance of nodes locally, while **betweenness centrality** (B), defined by counting the fraction of shortest paths passing through a given node [32], measures the global importance of nodes as information bridges in the network[24, 33]. The shortest path between two nodes is the path connecting two nodes with the least steps [34, 35].

The **Clustering coefficient** (C) is also an important metric to examine a network’s clustering feature, defined as the probability that two nodes both connected to a third node are also connected to each other.

**Community structure** is common for many real-world networks. Community detection attempts to find groups of nodes with dense internal connections and loose external connections. Researchers have proposed many algorithms to detect community structure [9, 17, 36]. We apply the modularity maximization approach [37] to detect the community structure of the global electricity trade network as it has the advantage of a faster run time.

### CO_2_ implications

Changes in CO_2_ emissions from electricity trade are calculated by:

$$E_{G}=\sum_{\left(i,j\right)}w_{ij}\left(EF_{i}-EF_{j}\right)$$ (1)

where *EF*_*i*_ and *EF*_*j*_ are CO_2_ emission factors of electricity generation in exporting country *i* and importing country *j*, respectively; *w*_*ij*_ is the volume of electricity exports from nation *i* to nation *j*; *E*_*G*_ is the total CO_2_ emission changes from electricity trade. A negative value of *E*_*G*_ indicates reduced CO_2_ emissions, while a positive value indicates an increase.

### Data sources

This study uses international electricity trade data from 1990 to 2010 from the UN Comtrade database (http://comtrade.un.org). Electricity trade is reported in both monetary ($) and physical units (MWh), though the early records in physical units are incomplete. We thus use data in physical units to conduct the network analysis for 2010 and monetary data to analyze the historical trend. We keep the trade data recorded by importing countries, and filtered certain unreasonable trade records. For example, Slovenia reportedly imported 3,182 MWk of electricity from Cyprus, which is beyond reasonable geographic extent. We deleted this record to avoid errors. After such data processing, the global electricity trade network covers 114 nations expressed by ISO 3 code abbreviations (S1 Table). We obtain CO_2_ emissions factors of electricity generation for each nation from the International Energy Agency [38].

## Results

### Global electricity trade network evolution

**Fig 1:**
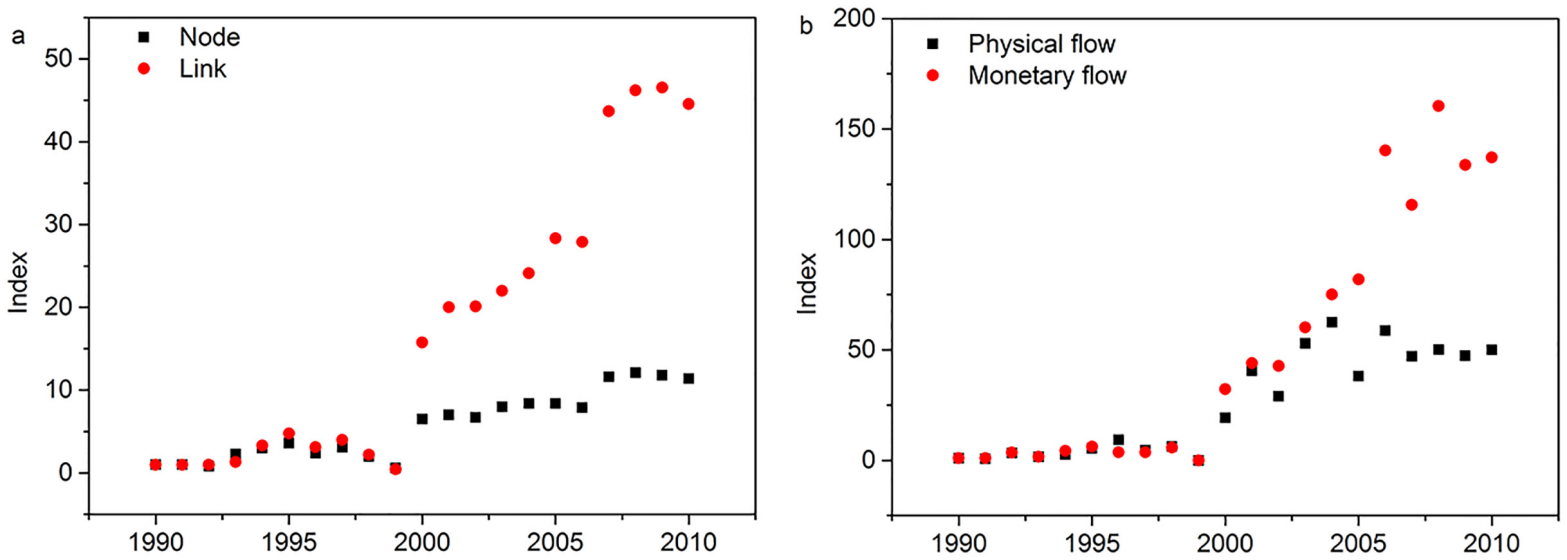
Dynamics of the global electricity trade network, 1990–2010. (a) Changes in number of nodes and links; (b) Changes in electricity trade volume. All data are normalized to the 1990 level.

The increasingly liberalized electricity market is evidenced by the fact that both the number of nations (nodes) and electricity trade volumes (links) increased during 1990–2010, from 10 nodes and 9 links in 1990 to 114 nodes and 400 links in 2010 (Fig 1), indicating increasing popularity of electricity trade between countries. Growth in the number of links outpaced that of nodes after 2000, implying increasing interconnectedness among countries. Electricity trade volume grows exponentially, increasing from 11.4 PWh (246.1 million $) in 1990 to 569.7 PWh (33.7 billion $) in 2010. The volume of electricity trade measured by value increased more rapidly after 2000 than that measured by physical trade. In particular, there is a shrink in 1999 due to the data unavailability for physical electricity trade.

Table 1 shows that the largest physical electricity flow increased from 6 PWh in 1990 to 46 PWh in 2010, while the mean flow remained relatively constant, suggesting increasing intensified electricity trade for a small number of nations.

**Table 1:** Properties of the global electricity trade network, 1990–2010.

Index	1990	1995	2000	2005	2010
No. of nodes	10	36	64	84	114
No. of links	9	43	141	255	400
Total electricity flow (PWh)	11	61	220	434	570
Largest electricity flow (PWh)	6	40	47	40	46
Mean electricity flow (PWh)	1	1	2	2	1
Largest in-degree	3	7	9	13	18
Largest out-degree	3	7	13	14	19
Largest node degree	6	14	20	24	34
Largest node strength (PWh)	11	42	68	88	151

### Global electricity trade network structure

**Fig 2:**
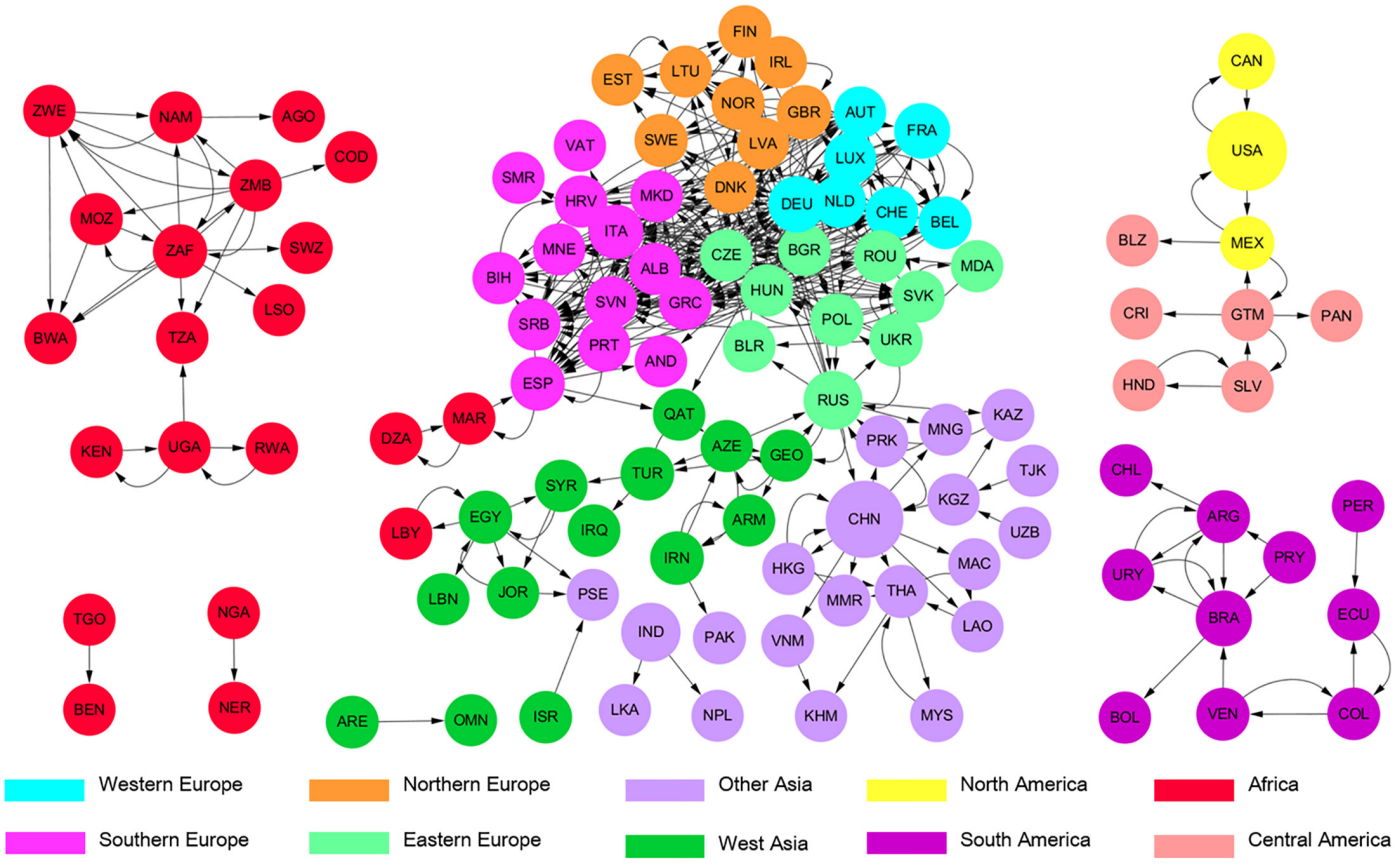
Global electricity trade network in 2010: African sub-network (left), Eurasian sub-network (middle), North and Central American sub-network (upper right), and South American sub-network (lower right). Link width indicates electricity trade volume, while node size represents the nation’s electricity exports. Full names corresponding to ISO 3 country codes are shown in S1 Table.

Geographical locations and transmission technologies significantly influence the structure of the global electricity trade network. The entire network can be divided into 4 sub-networks: African, Eurasian, South American, and North and Central American, Fig 2. The Eurasian sub-network covering 77 nations is the largest, with the most nations participating in electricity trade and the most intensive electricity trade (largest total electricity flow and mean node strength, Table 2). In addition, its mean clustering coefficient (0.29) is relatively high, indicating that electricity trading nations in the Eurasian sub-network have a high tendency to cluster and form tight trade groups. We focus the remaining analysis on the structure of the Eurasian sub-network.

**Table 2:** Properties of the four sub-networks in 2010.

Index	Eurasian Continent	Africa	North and Central America	South America
**No. of nodes**	77	18	9	10
**No. of links**	340	31	13	16
**Total electricity flow (PWh)**	465	21	69	14
**Mean node degree**	8.8	3.4	2.9	3.2
**Mean node strength (10**^**6**^ **MWh)**	12	2.4	15	2.8
**Mean clustering coefficient**	0.29	0.18	0	0.24
**Mean betweenness centrality**	0.03	0.01	0.13	0.09

#### Node degree and node strength

Tables 3 and 4 present the top 10 nations in the Eurasian sub-network in terms of node degree and node strength, respectively (Full list available in S2 and S3 Tables). The average nation in the Eurasian sub-network trades electricity with 8.8 partners, ranging from 1 to 34. Both the Czech Republic and Slovenia have the most trade partners (34), followed by Germany (31). Slovenia has the largest in-degree (18) value, while Austria has the largest out-degree (18) value. Node strength (the sum of exports and imports) measures a nation’s total electricity trade volume, ranging from 1 MWh to 151 PWh, averaging at 12 PWh. Germany has the largest node strength (151 PWh, mostly exports). Switzerland and Germany have the largest import and export strengths, indicating strong dependence on and by other countries, respectively.

**Table 3:** Top 10 Eurasian sub-network nations: node degree, 2010.

Rank	Out-degree		In-degree		Total node degree = out-degree + in-degree	
	Country	*k*_out_	Country	*k*_in_	Country	*k*
**1**	Austria	18	Slovenia	18	Czech Rep.	34
**2**	Czech Rep.	17	Czech Rep.	17	Slovenia	34
**3**	Germany	16	Germany	15	Germany	31
**4**	Slovenia	16	Switzerland	14	Austria	30
**5**	Switzerland	14	Greece	14	Switzerland	28
**6**	Hungary	13	Serbia	13	Hungary	24
**7**	Italy	13	Austria	12	Italy	24
**8**	Croatia	11	Croatia	11	Serbia	24
**9**	Russian Federation	11	Hungary	11	Greece	23
**10**	Serbia	11	Italy	11	Croatia	22

Notes: Full results available in S2 Table of the Supporting Excel file.

**Table 4:** Top 10 Eurasian sub-network nations: node strength, 2010 (Unit: PWh).

Rank	Export strength		Import strength		Total node strength = Export strength + Import strength	
	Country	*s*_out_	Country	*s*_in_	Country	*s*
1	Germany	100.0	Switzerland	70.6	Germany	150.8
2	France	75.8	Germany	50.8	France	95.2
3	Czech Rep.	26.7	Italy	49.9	Switzerland	86.5
4	Russian Federation	22.4	France	19.4	Italy	55.7
5	China	20.9	Austria	17.0	Czech Rep.	39.1
6	Austria	16.7	Hungary	15.9	Austria	33.7
7	Switzerland	15.8	Netherlands	15.7	Netherlands	29.6
8	Sweden	14.1	Finland	15.7	Sweden	29.0
9	Netherlands	13.9	Norway	15.1	China	26.5
10	Spain	13.8	Sweden	14.9	Belgium	25.6

Notes: Full results available in S3 Table of the Supporting Excel file.

Our results are consistent with practical situations. For example, Slovenia, net energy importer, imports about 14% of total electricity demand, mainly from Italy and Croatia. This explains it has the largest node in-degree. Czech Republic is the world’s fifth biggest power exporter and has few power imports. This explains why Czech Republic is ranked 3^rd^ in export strength but out of top 10 in import strength. Due to abundant renewable energy, Germany is the largest exporter with a net exporter by 20% during the year 2010, which makes it have the largest export strength. Austria, with approximately two thirds of the electricity generated provided by renewables, is net importer. This fact agrees our result that Austria ranks 5^th^ in import strength and 6^th^ in export strength (Table 4).

The link weights range from 1 MWh to 33 PWh, with a mean value of 1.3 PWh. The largest link in the Eurasian sub-network is electricity trade from Germany to Switzerland, accounting for 7% of the total electricity trade volume in this network (Table 5). The second largest link is from France to Switzerland, with 29 PWh and 6.1% of total trade volume. Refer to S4 Table for more information.

**Table 5:** Top 10 Eurasian sub-network nations: link weights, 2010 (Unit: PWh).

Export country	Import country	Quantity
Germany	Switzerland	32.51
France	Switzerland	29.03
France	Italy	21.34
Germany	Italy	16.54
France	Germany	14.34
Germany	Austria	14.30
Czech Rep.	Germany	12.56
Russian Federation	Finland	11.64
China	China, Hong Kong	11.11
Germany	Netherlands	8.94

Notes: Full results available in S4 Table of the Supporting Excel file.

**Fig 3:**
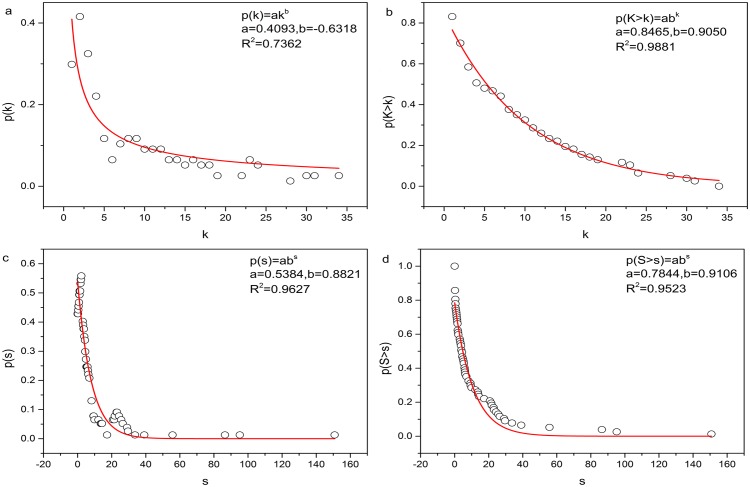
(a) Probability density of node degree; (b) cumulative density of node degree; (c) probability density of node strength; and (d) cumulative density of node strength. Black circles represent data, red lines represent fitted distributions.

Both probability density and cumulative density of node strength in the Eurasian sub-network follow the exponential distribution (Fig 3). The probability density of node degree follows the power law, while cumulative density of node degree follows the stretched exponential distribution (Fig 3).

#### Average nearest-neighbor degree and strength

**Fig 4:**
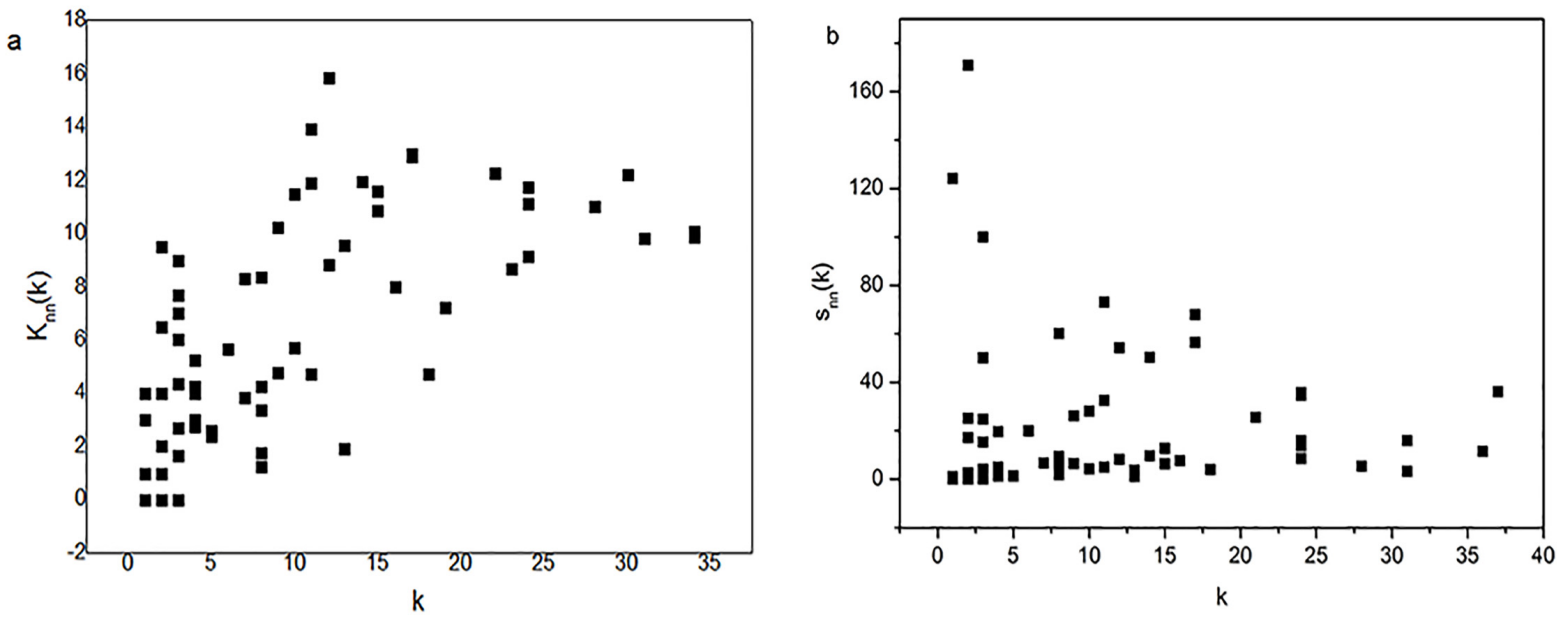
(a) Average nearest-neighbor node degree *K*_nn_ against node degree *k*; (b) average nearest-neighbor node strength *S*_nn_ against node degree *k*.

Here we use the average nearest-neighbor degree *K*_nn_ to analyze network assortativity, which measures similarity between connected nodes. If nodes with high degrees are more likely to connect to other nodes with high degrees, the network has the property of assortativity. Otherwise, if nodes with high degrees tend to connect to nodes with low degrees, the network is disassortative. Albania has the largest average nearest-neighbor degree at 15.3, followed by Bosnia-Herzegovina (14) and Bulgaria (13). Fig 4(a) shows no obvious correlation between average nearest-neighbor node degree *K*_nn_(*k*) and node degree *k*. In general, for most nodes with few trading partners, these partners may also have few trade neighbors. However, sometimes, a nation with many trading partners depends mainly on its partners’ neighbors. For example, China’s node degree is 13, and its average nearest neighbor is 3.3, while Albania’s node degree is 12 and its average nearest neighbor is 15.3. This is mainly because Albania trades with many other nations that in turn have many trading partners, like Greece and the Czech Republic. Fig 4(b) shows no obvious correlation between the average nearest-neighbor strength *S*_nn_(*k*) and node degree *k*, indicating that a nation in the Eurasian sub-network with few trading partners is likely to connect to nations with large electricity trade volumes.

#### Betweenness centrality and clustering coefficient

**Fig 5:**
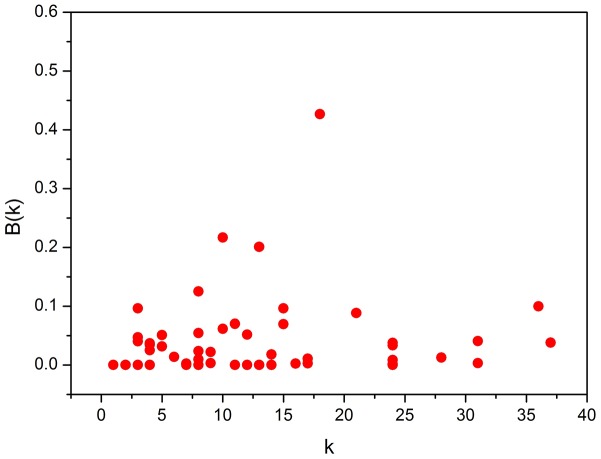
Node betweenness *B* against node degree *k*.

Nodes with high betweenness play a crucial role in a network by acting as bridges connecting other nodes [24, 33]. Russia, China, Ukraine, and Azerbaijan have the highest betweenness (Table 6 and S5 Table). These countries are thus critical to facilitating trade in the entire Eurasian electricity trade network, and are thus important for network stability. Fig 5 shows no obvious correlation between node betweenness and node degree. For example, Ukraine and Azerbaijan have small node degrees and node strengths, but high node betweenness. This indicates that, despite having few trading partners and less intensive electricity trade, these two nations are important facilitators in the entire Eurasian electricity trade network.

**Table 6:** Top 10 Eurasian sub-network nations: node betweenness and clustering coefficient, 2010.

Rank	Betweenness (B)		Clustering coefficient (C)	
1	Russian Federation	0.4227	Andorra	1
2	China	0.2018	Qatar	1
3	Ukraine	0.1863	China, Hong Kong	1
4	Azerbaijan	0.1257	Lao People's Dem. Rep.	1
5	Spain	0.0959	Rep. of Moldova	1
6	Mongolia	0.0943	Myanmar	1
7	Finland	0.0900	Mongolia	1
8	Slovakia	0.0860	Albania	0.8937
9	Norway	0.0791	TFYR of Macedonia	0.8930
10	Czech Rep.	0.0745	Bosnia Herzegovina	0.8631

Notes: Full results available in S5 Table of the Supporting Excel file.

The clustering coefficient *C* quantifies the local cliquishness of a network [12]. A higher clustering coefficient for a node indicates dense interconnectedness among its neighbors. The mean value of *C* for the Eurasian electricity trade network is 0.3507. The clustering coefficient of Andorra, Qatar, China Hong Kong, Lao People’s Dem. Rep, Moldova, Myanmar, and Mongolia are the highest at 1 (Table 6, S5 Table), implying that the probability that two of its neighbors are linked is 100%, in other word, all of its neighbors connected.

#### Communities

A community in the electricity trade network consists of a group of nations tightly connected by electricity trade. Changes in one nation have more effects on nations within the same community than nations outside the community. The community structure of the electricity trade network provides the foundation for assessing the impacts of cascading failures and intentional attacks on its stability.

**Fig 6:**
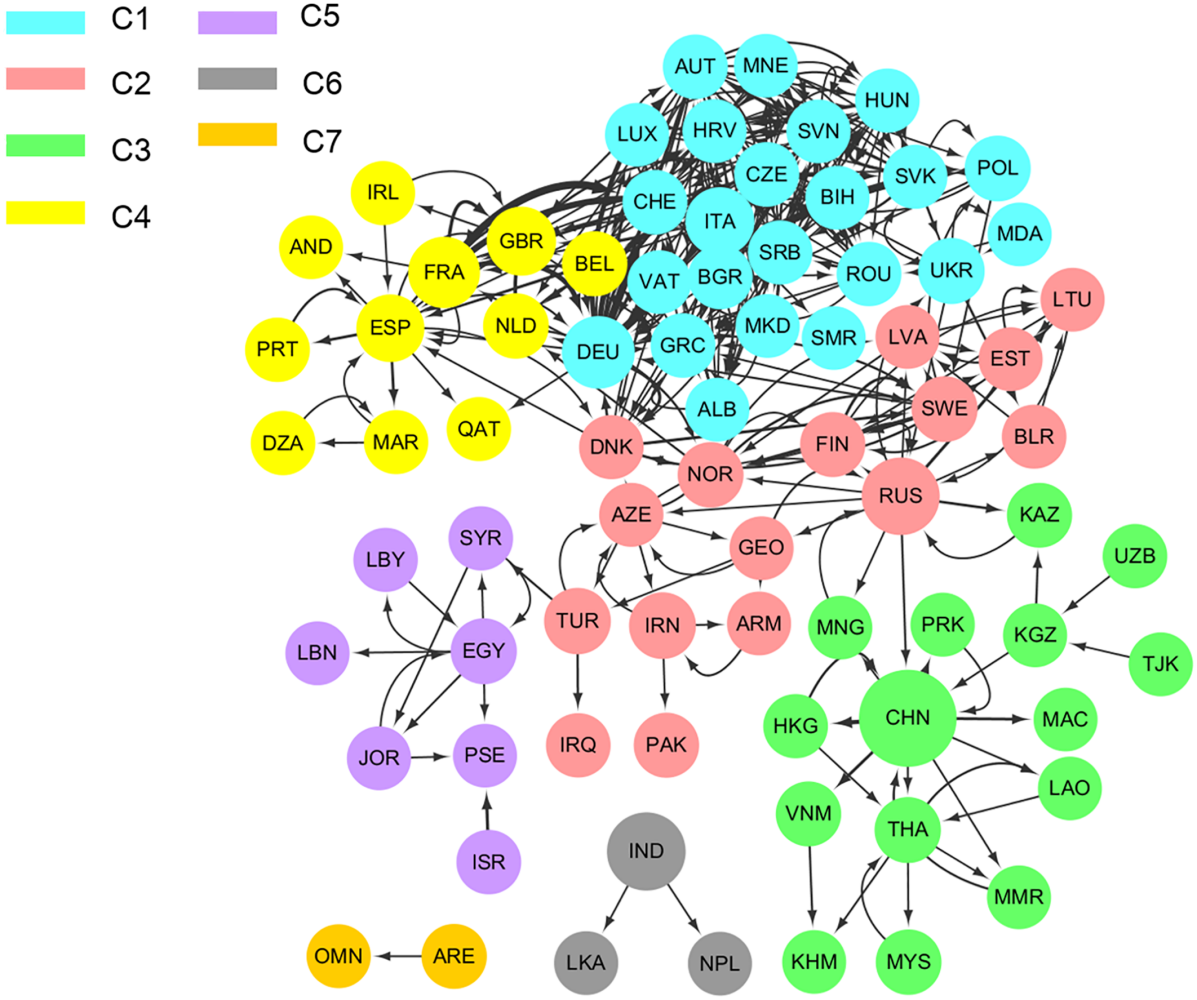
Eurasian sub-network community structure. Full names corresponding to ISO 3 country codes are shown in S1 Table.

The Eurasian sub-network has seven communities (Fig 6 and Table 7). The largest community (*C1*) has 23 nations connected by 202 links. The smallest community (*C7*) consists of the UAE and Oman, both of which lack electricity trade with other countries. India, Sri Lanka, and Nepal form an isolated three-node community, *C6*.

**Table 7:** Eurasian sub-network communities, 2010.

Community index	No. of nations	Description
C1	23	Albania, Austria, Bulgaria, Bosnia Herzegovina, Switzerland, Czech Rep. Germany, Greece, Croatia, Hungary, Italy, Luxembourg, Moldova, Macedonia, Montenegro, Poland, Romania, San Marino, Serbia, Slovakia, Slovenia, Ukraine, Holy See
C2	16	Armenia, Azerbaijan, Belarus, Denmark, Estonia, Finland, Georgia, Iran, Iraq, Lithuania, Latvia, Norway, Pakistan, Russia, Sweden, Turkey
C3	15	China, China-Hong Kong, Kazakhstan, Cambodia, Lao People's Dem. Rep., China-Macao, Myanmar, Mongolia, Malaysia, People’s Rep. of Korea, Thailand, Tajikistan, Uzbekistan, Viet Nam
C4	11	Andorra, Belgium, Algeria, Spain, France, United Kingdom, Ireland, Morocco, Netherlands, Portugal, Qatar
C5	7	Egypt, Israel, Jordan, Lebanon, Libya, State of Palestine, Syria
C6	3	India, Nepal, Sri Lanka
C7	2	United Arab Emirates, Oman

Notes: Full results available in S6 Table of the Supporting Excel file.

Geographical proximity plays an important role in community formation, as short distances make it easier to build transmission lines, but community structures show that this is not the only factor. Electricity trade among nations are also influenced by other factors such as political relationships and the landscape influencing grid construction costs (e.g., mountains versus flatlands). For example, Kazakhstan, is located near Russia but belongs to different communities. This may because that Kazakhstan is a bi-continental country and has once belonged to Former Soviet Union. Although there is electricity trade relationship between Russia and Kazakhstan, Russia has more frequent trade relationship with other European countries. The diplomacy relationship between them may be also an influence factor. Moreover, Pakistan is located near China and has strong economic interactions with it, but they belong to different communities in the Eurasian electricity trade network. This mainly due to the topography features obstacle the power facilities construction between China and its western neighbors. One the one hand, the electricity industry in sparsely populated Tibet in West China is poor. On the other hand, the Himalayas on the border is the natural Barrie. Thus, the community structure of the Eurasian electricity trade network reveals new interdependence relationships among nations. The countries in the same cluster have closer and stronger relationships with one another. Friendly diplomatic and favorable geographical conditions contribute a lot to reach an agreement on allocation mechanism and power facilities construction. Cross-board electricity trade benefits resource allocation on a larger scale, especial with the increasing development of unstable renewable energy generation.

The within-community degree z-score (Table 8) quantifies how well-connected a node is to other nodes within the community [12, 39]. A higher z-score indicates a greater importance in the community’s formation. S6 Table shows the nations with the highest z-score in each community. For example, China (2.9795) and Spain (2.6954) are the most important in the formation of communities *C3* and *C4*, respectively.

**Table 8:** Most important nations and z-scores in each community, 2010.

Community index	No. of nations	Links within community	Links among communities	Most important nation	z-scores
C1	23	202	43	Czech Republic	1.7481
C2	16	48	27	Russia	2.1412
C3	15	23	5	China	2.9795
C4	11	22	30	Spain	2.6954
C5	7	11	1	Egypt	1.9597
C6	3	2	0	India	1.4142
C7	2	1	0	-	-

Notes: Full results available in S6 Table of the Supporting Excel file.

### CO_2_ implications of electricity trade

**Fig 7:**
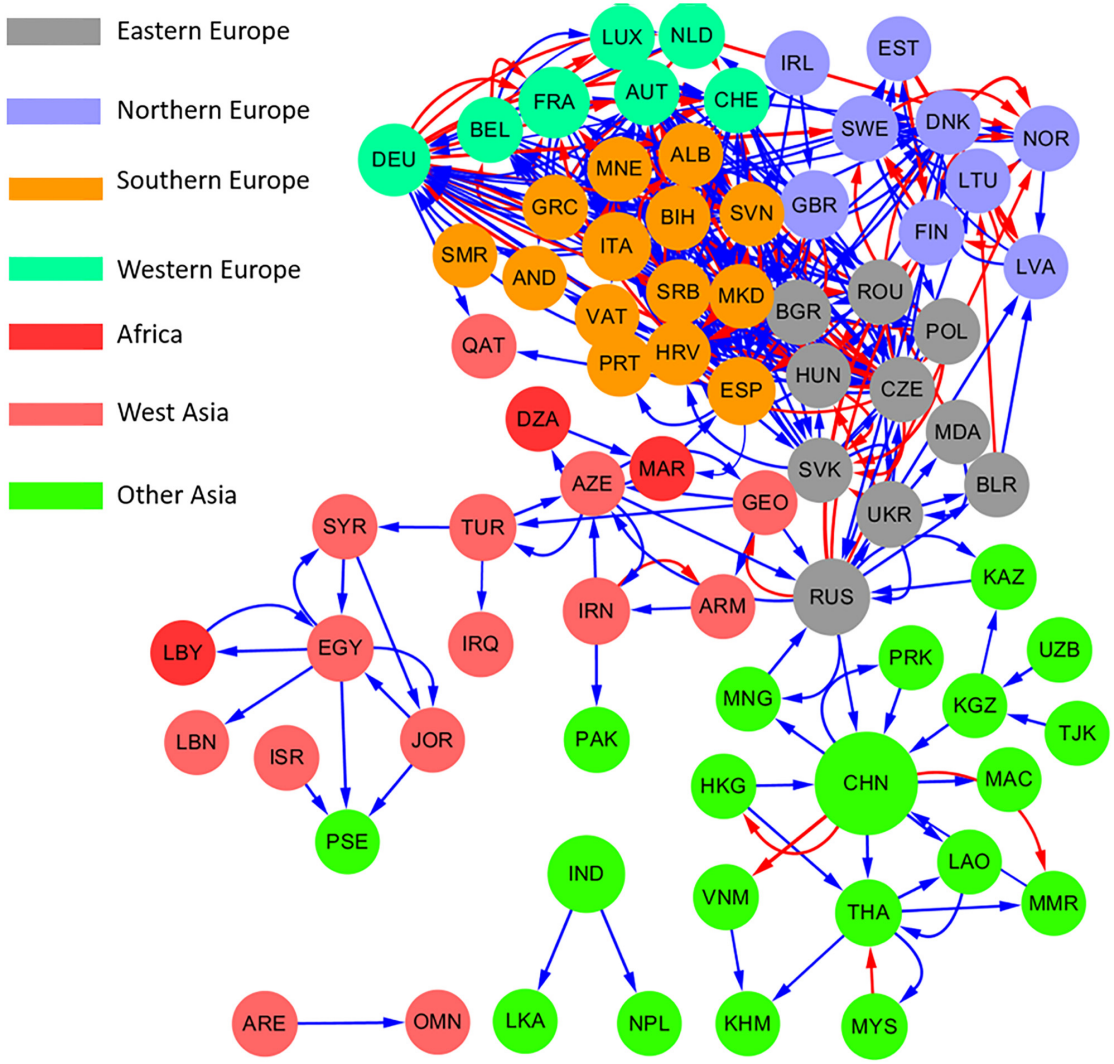
CO_2_ implications of electricity trade in the Eurasian sub-network. The direction of links reflects the flows of electricity trade; the width of links is proportional to the change of CO_2_ emissions due to electricity trade; and the color of links represents the effect of electricity trade on CO_2_ emissions (red for reduction and blue for increase). Full names corresponding to ISO 3 country codes are shown in S1 Table.

Fig 7 shows the CO_2_ implications of electricity trade in the Eurasian sub-network in 2010. Nations can reduce its CO_2_ emissions by importing electricity from other countries. For example, Albania has the lowest CO_2_ emission factor (2 kg/MWh) due to the significant share of renewable energy sources. Moreover, Albania exports 365 GWh of electricity to Greece, which has a much higher CO_2_ emission factor (718 kg/MWh). For Greece, importing less CO_2_-intensive electricity from Albania can reduce its own generation of CO_2_-intensive electricity, thus reducing CO_2_ emissions by approximately 0.3 million tons (Mt). However, international electricity trade in the entire sub-network actually increases CO_2_ emissions (11.0 Mt more) compared to a scenario where all countries produce their own electricity.

To date, the primary goal of international electricity trade is economical, cost-efficient electricity supply, with little attention paid to environmental issues, such as CO_2_ emissions. With increasingly stringent environmental regulations targeting the power sector [40, 41], especially in the European Union, one might expect to see more exports of “cleaner” electricity and less exports of “dirty” electricity, thus favoring the production of less emission-intensive electricity over emission-intensive electricity. It is no longer proper to evaluate the actual CO_2_ emissions associated with the terminal electricity consumption by calculating the local production- or consumption-based emission factors due to CO_2_ emissions from the electricity trade. Further, “carbon leakage” [42], where emission-intensive electricity produced in countries without stringent regulations may gain favor without universal regulations for an interconnected electricity trade network, could occur.

## Discussion and Conclusion

This is the first study offering an analysis of the structure of the global electricity trade network consisting of four sub-networks: African, Eurasian, South American, and North and Central American. As the largest sub-network, this study uses the Eurasian sub-network as an example to identify critical nations in the global electricity trade network using various metrics. Cross-border electricity trade is intensive in Europe. Germany, France, and the Czech Republic are the largest electricity exporters, and the reliability of their national grids is important to downstream partners. Cross-border electricity trade can take full advantage energy, especially renewable energy generation. Those great net electricity importers (e.g. Italy) may face significant energy security issues. They should thus aim to diversify their sources and adopt long-term cooperation strategies to guarantee electricity supply security.

Russia, Ukraine, China, and Azerbaijan have more central positions as measured by betweenness centrality. They are major bridges connecting intensive European communities with less active Asian communities in cross-border electricity trade. They also play an important role in the security of Eurasian sub-network from the overall view. Due to the diplomacy relationship and the geomorphological conditions, communities in the Eurasian sub-network do not fully align with geographical proximity. Moreover, the present international electricity trade in this sub-network creates an approximately 11 million additional tons of CO_2_ emissions in 2010. This analysis shows that electricity trade networks could also be used to analyze other environment influences from global electricity trading.

There are many other index in network analysis, e.g. motif, scale-free feature, distance of networks, cliques, matching, dominating sets, degree assortativity coefficient, degree Pearson correlation coefficients, degree mixing matrix, edges weighted of networks. This work is the first attempt to analyze global electricity trade network. In our future study, more network analysis indexes will be adopted, and deeper policy implication will be explored.

## Supporting Information

S1 Table ISO 3 country code abbreviations. (XLSX)

S2 Table Node degree of nations in Eurasian sub-network in 2010. (XLSX)

S3 Table Node strength of nations in Eurasian sub-network in 2010. (XLSX)

S4 Table Link weights in the Eurasian sub-network in 2010. (XLSX)

S5 Table Node betweenness and clustering coefficient of nations in Eurasian sub-network in 2010. (XLSX)

S6 Table Communities and z-scores of nations in the Eurasian sub-network in 2010. (XLSX)
